# Keep on walking

**DOI:** 10.2807/1560-7917.ES.2022.27.2.2201131

**Published:** 2022-01-13

**Authors:** Eurosurveillance editorial team

**Affiliations:** 1 European Centre for Disease Prevention and Control (ECDC), Stockholm, Sweden

At the beginning of 2022, as we head into the third pandemic year, we would like to take the opportunity to acknowledge the hard work of all those who have been involved in public health and infectious disease prevention and control over the past 2 years. We also express our gratitude to our contributors, supporters and readers to whom we send our best wishes for the weeks and months ahead. 

As in the past, we start the year by providing a brief overview of our activities during the previous year and give an outlook on some planned developments for 2022.

Continuing from 2020, coronavirus disease (COVID-19) predominated among the topics covered in *Eurosurveillance *also in 2021. Not surprisingly, the 10 most viewed articles were all on COVID-19 and the top two, a research paper by Scheiblauer et al. and a rapid communication by Brandal et al. were published only recently, in November and December. However, we also aimed to raise awareness of issues related to two other respiratory diseases: the widespread physical distancing measures and restricted movements of people to curb the pandemic led to a near total absence of influenza and RSV infections during their usual seasons in 2020 and 2021. As non-pharmacological interventions were gradually lifted, we ran a focussed issue in July, describing the return of RSV infections with notably different seasonal patterns and the possible public health impact of this, as well as a ‘Spotlight on influenza’ series in September/October that highlighted possible challenges for the upcoming northern hemisphere season 2021/22. As usual, we covered important health days such as World Tuberculosis Day in March, European Antibiotic Awareness Day in November and World AIDS Day in December. We also undertook a series of activities to highlight our 25th anniversary. To mark our aspirations over a quarter of a century, we chose the theme ‘25 years of public health impact’, set up a dedicated page with a special collection, and links to presentations illustrating the journey, achievements and public health impact of the journal in the past and present. Our anniversary seminar dedicated to ‘Epidemiology and immunology – from observation to explanation and public health action’ was well received and we thank our international speakers who covered aspects of influenza, measles and COVID-19, for their interesting presentations.

We published 207 articles (87 rapid communications, 107 regular articles and a total of 13 editorials and letters). On average we received 72 submissions per month, amounting to 1,241 submitted articles, and we accepted one in six of these (acceptance rate: 16%) for publication. Once again, about 500 experts from all across the world dedicated their time to guide us in the decision-making process by sharing their views and comments on articles. Being aware of the pandemic’s strain on the experts in our field, we especially appreciate their support and dedication in the last year. A list with their names can be found in our first 2022 issue. We wish to extend our gratitude to the many unnamed people who have supported us in different ways also in 2021, and we thank in particular our board members for their dedication and providing strategic and hands on guidance, our contributors for their trust in the journal, our supporters and colleagues at ECDC for providing invaluable advice as well as our publisher and ECDC Director, Andrea Ammon, for providing important resources and granting us editorial independence. We could not have done our work without their support and encouragement.

COVID-19 will remain an important topic in 2022. Nevertheless, we will cover various other topics and together with our board, we identified ‘Food as a vehicle for antimicrobial resistance’ as an annual theme to raise awareness and to guide our selection of articles in 2022. The theme encompasses a wide range of aspects such as general surveillance in humans and animals at regional, national and international level, antimicrobial use and resistance in farmed animals, including aquaculture, the risk from potentially contaminated food items that are usually consumed raw, behavioural aspects such as awareness of risky food and eating habits, the possible development of simple and rapid testing to detect AMR in food, the environment as intermediate or direct source of resistant infections in animals or humans, as well as targeted prevention and control efforts based on available evidence and the role of international organisations in supporting countries. We are looking forward to receiving interesting contributions on this important topic.

With respect to new initiatives in 2022, we would like to announce two coming up soon. The first aims to support capacity building and provide recognition to peer reviewers by promoting the involvement of early career researchers in peer review. The second initiative pays tribute to the fact that content in scientific articles has, particularly during the pandemic, provided the evidence for rapid policy-making and featured in the media nearly on a daily basis. This has highlighted the importance of communicating key findings in a clear manner. The *Eurosurveillance* editorial team will start a pilot during the first quarter of 2022, working with authors to extract key public health messages from their articles to enhance the potential impact of their work. 

At the beginning of the new year, we are aware of the persisting high demands on all those concerned with public health and communicable diseases prevention and control. We will continue to support their work through selecting articles that provide authoritative data, evidence and guidance to facilitate the implementation of effective prevention and control measures, and to support the preparedness and response to health threats in Europe. We look forward to doing this jointly with our contributors and many other supporters.

